# *Sporomusa ovata* as Catalyst for Bioelectrochemical Carbon Dioxide Reduction: A Review Across Disciplines From Microbiology to Process Engineering

**DOI:** 10.3389/fmicb.2022.913311

**Published:** 2022-06-20

**Authors:** Joana Madjarov, Ricardo Soares, Catarina M. Paquete, Ricardo O. Louro

**Affiliations:** ^1^Instituto de Tecnologia Química e Biológica António Xavier, Universidade Nova de Lisboa, Oeiras, Portugal; ^2^Instituto Nacional de Investigação Agrária e Veterinária, Oeiras, Portugal

**Keywords:** microbial electrosynthesis, *Sporomusa ovata*, electron uptake mechanisms, microbial cathodes, *c*-type cytochromes

## Abstract

*Sporomusa ovata* is a bacterium that can accept electrons from cathodes to drive microbial electrosynthesis (MES) of acetate from carbon dioxide. It is the biocatalyst with the highest acetate production rate described. Here we review the research on *S. ovata* across different disciplines, including microbiology, biochemistry, engineering, and materials science, to summarize and assess the state-of-the-art. The improvement of the biocatalytic capacity of *S. ovata* in the last 10 years, using different optimization strategies is described and discussed. In addition, we propose possible electron uptake routes derived from genetic and experimental data described in the literature and point out the possibilities to understand and improve the performance of *S. ovata* through genetic engineering. Finally, we identify current knowledge gaps guiding further research efforts to explore this promising organism for the MES field.

## Introduction

**FIGURE 1:**
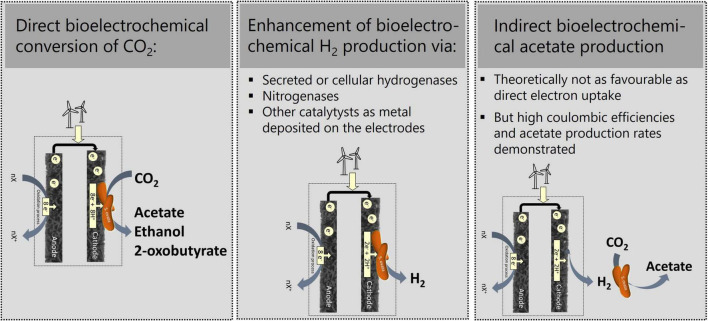
Basic principles and versatility of microbial electrosynthesis with *S. ovata.*

Electrotrophs such as *Sporomusa ovata* are organisms capable to use electrons collected from electrodes as an energy source. Together with the autotrophic capacity to produce complex organic compounds from carbon dioxide (CO_2_), these capabilities enable the production of various chemicals in a process called microbial electrochemical synthesis (MES; Jourdin and Burdyny, 2020). Given that MES allow the production of chemicals from CO_2_ and renewable energy (Figure 1), it promotes the reduction of CO_2_ emissions and enables the implementation of a circular economy.

The main products of MES reported so far are acetic acid, ethanol, *n*-butyric acid, *n*-butanol, hexanoic acid, and *n*-hexanol (Jourdin and Burdyny, 2020). Among these, acetate represents the product in 75% of all studies (Flexer and Jourdin, 2020). The worldwide industrial acetate production reached 3.5 million tons (Jourdin et al., 2020) and despite the political efforts for climate change mitigation by decreasing petroleum usage, 90% are still produced from fossil oil and gas (Vidra and Nemeth, 2018). The main importance of acetate is its role as C2 platform chemical used to generate a plethora of different products in the organic chemistry industry. Starting from acetate, e.g., acetic anhydride can be produced by chain elongation which is subsequently converted to cellulose acetate. Cellulose acetate is used to produce textile fibers, cigarette filters, and plastics (Bajracharya, 2016; Falcke et al., 2017).

Koch and Harnisch (2016) identified 45 bacterial strains capable of cathodic electron uptake, with 13 being also autotrophic and thus candidates for MES. Das et al. (2020) rated the acetate production of different pure cultures, showing that *S. ovata* is by far the most promising biocatalyst. It produces 51.1 g m^–2^ day^–1^ of acetate compared to 12.8 g m^–2^ day^–1^ by the second best, *Acetobacterium woodii*, and 0.14 g m^–2^ day^–1^ by *Clostriudium ljungdahlii*. There is already a genetic system and fundamental mechanistic studies *of C. ljungdahlii* and *Acetobacterium ferrooxidans* (Sydow et al., 2014), but the production rates of valuable products are not competitive compared to *S. ovata*. *S. ovata* is reported to be the best acetate producer when used as a pure culture. The highest reported current density with *S. ovata* is 22 A m^–2^ (Aryal et al., 2019).

The main requirements to achieve the needed transition of MES to industrial-scale are the improvement of (i) conversion rates, (ii) electron uptake rates, (iii) chemical production rate, and (iv) product selectivity (Flexer and Jourdin, 2020). Research aiming to close these gaps is intense, highly dynamic, and frequently reviewed. The current state-of-the-art and future perspectives of MES, in general, were recently described (Bian et al., 2020; Jourdin and Burdyny, 2020; Prévoteau et al., 2020; Dessì et al., 2021), its techno-economic future assessed (Jourdin et al., 2020), and a ranking regarding its Technology Readiness Level provided (Fruehauf et al., 2020). These show a solid basis of knowledge about MES. Among others, several non-biological operation parameters such as temperature, salinity, and pressure are elaborated as necessary and easily attainable optimization parameters (Jourdin and Burdyny, 2020). Furthermore, selecting electrode materials and architecture optimized toward mass transport and current distribution inside the structure shows a high potential to increase product yields (Jourdin and Burdyny, 2020). But the core electron uptake process of the microorganisms catalyzing the desired reaction, also recently reviewed in Karthikeyan et al. (2019), is still unknown to a great extent. Exemplary, it is becoming widely accepted that indirect electron transfer is predominant compared to the direct electron uptake from an electrode (Prévoteau et al., 2020). The indirect electron transfer is proposed to occur *via* hydrogen produced with the help of secreted hydrogenases or catalytically active precipitates on the electrodes (Tremblay et al., 2019), but their nature is not conclusively clarified.

Whether *S. ovata* uses direct or indirect electron transfer has a decisive impact on tailoring the design of a reactor, electrode materials, and the process parameters. If indirect electron transfer *via* hydrogen is dominant, it will be essential to understand how *S. ovata* achieves the necessary hydrogen evolution rates at high electrode potentials. This knowledge will push related fields such as green hydrogen production forward, as presented in Figure 1. Furthermore, it will help to decide on two-stage or one-stage reactors. In the case of indirect electron uptake, two-stage reactors can be used where hydrogen production and the subsequent conversion of hydrogen and CO_2_ to acetate can be optimized separately. A two-stage reactor in turn will detain the possibility of the supportive effects on hydrogen evolution by the presence of *S. ovata* seems to have (Tremblay et al., 2019). Thus, the development of the optimal operational conditions relies on elucidating the fundamental mechanism of indirect electron transport. If, by contrast, direct electron transfer is dominant, then the responsible biochemical pathway remains to be identified. From the genome, we expect electron uptake mechanisms to be different from those described for electrotrophs already studied at the biochemical level, such as *Rhodopseudomonas palustris* TIE-1 or *Rhodobacter ferrooxidans* SW2 (Saraiva et al., 2012; Bird et al., 2014). Direct electron uptake would enable the use of one-stage reactors, which show a smaller operational complexity but need to meet the requirements of *S. ovata* and be optimized toward electrochemical parameters at the same time. Thus, fundamental research on the electron transfer mechanisms between the cathode and the biocatalyst is sorely needed.

Within this review, we suggest studying *S. ovata* as a model organism to enable the detection of bottlenecks and further development of MES. Understanding the molecular mechanisms of electron uptake of *S. ovata* will lead to the needed knowledge to steer the work in this area.

## The Genus *Sporomusa*

Bacteria of the genus *Sporomusa* are strictly anaerobic, stain Gram-negative, and form spores. These two latter properties are unusual to be found together, and despite staining Gram-negative, the genus belongs to the Phylum of Gram-positive bacteria (Stackebrandt et al., 1985). A *Sporomusa* isolate is typically identified by the size between 0.4–0.9 × 2–8 μm, the motility, the banana shape, the formation of endospores, the true Gram-negative character with the presence of two membranes, and the production of acetic acid (Moeller et al., 1984). Substrates that all *Sporomusa* species can metabolize are *N*-methyl compounds, hydroxy fatty acids, primary alcohols, and a mixture of H_2_ and CO_2_, with the typical fermentation product being acetate (Moeller et al., 1984). *Sporomusa* species were isolated from various anoxic environments such as soils, sediments, digestive systems, and wastewater (McClane et al., 2006).

*Sporomusa* species perform homoacetogenic metabolism, which means that they can form acetate from CO_2_ and H_2_ *via* the acetyl-CoA pathway for energy conservation (also known as Wood Ljungdahl, WL-Pathway). This pathway is an ecologically important way to fix CO_2_, being identified in more than 100 bacteria and is expected to account for a share of approx. 20% of the worldwide carbon fixation (Ljungdahl, 2009).

### The Metabolism of *S. ovata*

**FIGURE 2:**
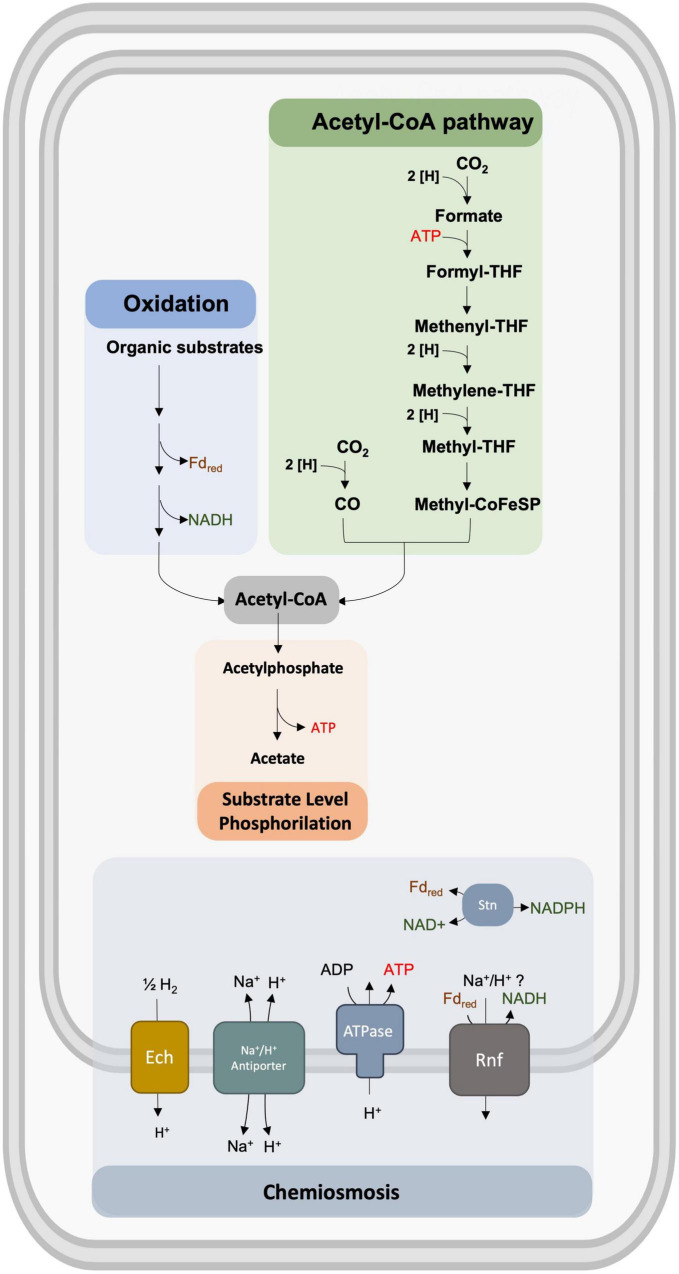
Representation of the bioenergetic metabolism of *S. ovata* that includes the acetyl-CoA pathway, the oxidation of organic substrates, the generation of ATP by substrate-level phosphorylation (SLP), and chemiosmosis.

The species *S. ovata* was first described in 1984 by the work of Moeller et al. (1984), where ovata was named due to its egg-shaped endospores. A critical feature of *S. ovata* enabling the assimilation of CO_2_ is its homoacetogenic nature that uses the reductive acetyl-CoA pathway for energy conservation and CO_2_ fixation (Visser et al., 2016). The acetyl-CoA pathway contains two branches that allow the conversion of two molecules of CO_2_ to acetyl-CoA (Schuchmann and Müller, 2016). In the methyl branch, CO_2_ is reduced to formate, which is subsequently bound to the cofactor tetrahydrofolate, being reduced step wisely to become a precursor of the methyl group of acetyl-CoA. In the carbonyl branch, a second molecule of CO_2_ is reduced to CO to become the precursor for the carbonyl group of acetyl CoA, catalyzed by the carbon monoxide dehydrogenase/acetyl-CoA synthase (CODH/Acs; Schuchmann and Müller, 2016). Acetyl-CoA can then be incorporated into biomass or converted to end-products, including acetate. Interestingly, the role of this pathway is to provide an electron sink for redox balancing instead of feeding a respiratory chain. Indeed, no net ATP is gained when acetate is produced, given that one mole of ATP is consumed in the acetate kinase reaction, and one mole of ATP is produced in the formyl-tetrafolate synthetase reaction (Figure 2). The advantage of this pathway is that it can be easily interfaced with a vast variety of electron donors, allowing acetogens to utilize energy from several substrates available in anoxic environments. Indeed, coupling acetogenesis to the conversion of heterotrophic substrates results in improved energy gain compared to classic fermentation (Müller, 2003). *S. ovata* can use H_2_ together with one-carbon compounds, including CO_2_, formate, methanol, and *N*-methylated compounds, but can also use betaine, sarcosine, fructose, lactate, pyruvate, ethanol, *n*-propanol, *n*-butanol, 2,3-butanediol, L-Alanine, and 1,2-propanediol as substrates (Visser et al., 2016).

All genes coding for enzymes of the acetyl-CoA pathway are present in the genome of *S. ovata* (Kremp et al., 2020). All these enzymes are predicted to be soluble cytoplasmic proteins, with none of them being involved in chemiosmotic energy conservation (Schuchmann and Müller, 2016). Therefore, the reducing equivalents in these organisms are gained by the oxidation of H_2_ during autotrophic growth or by NADH and reduced ferredoxin during heterotrophic growth. Membrane-integral protein complexes then oxidize these reduced compounds to generate a chemiosmotic gradient that is used for ATP generation (Figure 2). Acetogens differ in the respiratory enzymes used. While some use a sodium ion-translocating ferredoxin:NAD + oxidoreductase (Rnf) or the ion-translocating ferredoxin:H + oxidoreductase (Ech), others use an NADH-dependent reduced ferredoxin:NADP + oxidoreductase (Nfn; Schuchmann and Müller, 2014, Schuchmann and Müller, 2016; Kremp et al., 2020). Interestingly *S. ovata* has genes encoding for the Rnf complex and genes encoding the Ech Complex (Müller, 2019). Rnf complexes couple the electron transfer from reduced ferredoxin to NAD + to the translocation of sodium ions or protons across the cytoplasmic membrane, contributing to chemiosmotic ion translocation and thus ATP synthesis (Hess et al., 2013; Tremblay et al., 2013; Kremp et al., 2020; Figure 2). This is only possible by electron bifurcation, which is a mechanism that couples endergonic to exergonic redox reactions, allowing to save cellular ATP (Kremp et al., 2020). However, the ability of *S. ovata* to use both NADH and NADPH as reductants in the acetyl-CoA pathway and the absence of *nfn* genes in its genome led to the identification of a novel *Sporomusa* type Nfn (Stn; Kremp et al., 2020). This complex, composed of existing modules of the soluble Fe–Fe hydrogenase and Nfn, links the cellular NADP(H) pool with the NAD(H) and the ferredoxin pool. It is responsible to catalyze the energetically downhill reaction of NAD + or NADP + reduction by reduced ferredoxin and the reduction of NAD + with NADPH (Kremp et al., 2020).

### Laboratory Growth Conditions

*Sporomusa ovata* can be grown in the DSMZ medium 311 with betaine as substrate but has also been cultivated with H_2_ and CO_2_. The optimal temperature to grow the mesophilic *S. ovata* is between 34 and 39°C (Moeller et al., 1984), and the optimum pH range lies between 5.3 and 7.2 (Moeller et al., 1984). Many acetogens including *Sporomusa sp.* can grow on iron [Fe(0)] as an electron donor, but not *S. ovata* (Kato et al., 2015). Similar to the cathodes in MES, Fe(0) is a solid electron donor. It is surprising, that *S. ovata* cannot use it to take up electrons.

*Sporomusa ovata* grown on cathodes use the electrode as an electron source and CO_2_ as the carbon source to sustain their autotrophic metabolism. We found that pre-cultures for bioelectrochemical experiments are also grown autotrophically under H_2_ and CO_2_ conditions (Aryal et al., 2016, 2017b,^2018^, ^2019^; Chen et al., 2016). We have not found any published study inoculating bioelectrochemical experiments directly from a betaine grown *S. ovata* culture.

An investigation of the effect of medium salinity on the growth of *S. ovata* shows, that an increase of the NaCl concentration significantly affects the doubling time: it increases from 10 to approx. 25 ± 5 h when the ionic strength is modified from 100 to 200 mM NaCl (Sakimoto et al., 2014). Interestingly, this study also demonstrated that increased salinity induces filamentous growth and the alignment of the cells on a photoactive silicon nanowire. The hydrostatic pressure in the growth conditions has a smaller impact: the doubling time is around 15 h at 500 kPa, while it is approx. 5 h faster at only 100 kPa (Sakimoto et al., 2014).

## Optimization Strategies Aiming to Enhance Bioelectrochemical Acetate Production Using *S. ovata*

The first published work with *S. ovata* producing acetate on a cathode was performed by Nevin et al. (2010). Since then, *S. ovata* has been continuously used and different strains, electrode materials, operating parameters, and process concepts were evaluated with the aim to increase the acetate production rate. The acetate production rate normalized to the electrode surface is a parameter that allows the assessment of the quality of the electrobiocatalyst. Other important parameters such as volumetric production rates, coulombic efficiencies, product concentration, and energetic efficiencies are crucial for the overall assessment of MES concepts and their upscaling potential. However, they also relate to the reactor engineering, operation parameters, and experimental setup, which are not in the main scope of this review. Also beyond the scope of this review is the important work being done to explore how the production of other compounds such as alcohols can be manipulated (Ammam et al., 2016). So, to focus on *S. ovata* and stick to the aimed acetate production, we chose the normalized production rate as the parameter to establish a common denominator to compare different studies.

### Microbiology

The biocatalyst plays a leading role regarding the aimed cathode reactions. Thus, engineering *S. ovata*, adapting it, or selecting suitable strains, directly influences the acetate production rate.

#### Strain Selection

**FIGURE 3:**
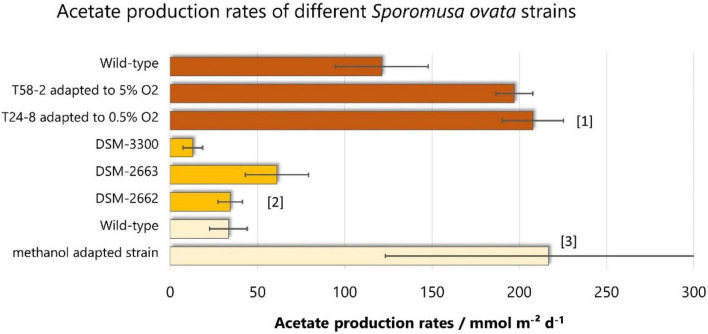
Acetate production rates of different *S. ovata* strains. Bars of the same color are derived from the same studies: [1]: (Shi et al., 2021), [2]: (Aryal et al., 2017b), [3]: (Tremblay et al., 2015). On that basis, results of the same color are directly comparable, as other parameters (cathode potential, medium, biofilm immobilization, reactor configuration) are not altered.

There are only a few studies directly comparing different pure cultures for electroacetogenesis. One of them reveals that different *Sporomusa* species produce different amounts of acetate (Aryal et al., 2017b): *S. aerivorans* does not produce any detectable acetate, while *S. ovata* DSM-2663 outcompetes all other tested species or strains (*S. malonica* DSM-5090 and *S. aerivorans* DSM-13326, *S. acidovorans, S. ovata* DSM-2662, *S. ovata* DSM-3300) with respect to acetate production rate (61.1 ± 18.1 mmol m^–2^ day^–1^) (see Figure 3). Regarding coulombic efficiency, *S. ovata* DSM-2662 showed the best results, with 91.8% ± 5.3 compared to 61.1 ± 12.6% when using *S. ovata* DSM-2663. Another study shows that *S. ovata* clearly outcompetes *Moorella* species: 7.6 ± 0.2 mmol m^–2^ day^–1^ for *S. ovata* vs. 2.1 ± 0.2 mmol m^–2^ day^–1^ for *M. thermoacetica* and 3.5 ± 0.3 mmol m^–2^ day^–1^ for *M. thermoautotrophica* (Faraghiparapari and Zengler, 2017).

#### Adapted Laboratory Evolution

So far, no genetic engineering of *S. ovata* was reported. But adapted laboratory evolution (ALE) experiments show a great potential to improve the acetate production of *S. ovata* at the microbiological level. With ALE experiments adapting *S. ovata* to methanol, Tremblay et al. (2015) significantly increased the acetate production rate by 6.5-fold. The adapted strain produced 866.7 ± 373.5 mM m^–2^ day^–1^ at -690 mV vs. SHE, compared to 133.5 ± 43.2 mM m^–2^ day^–1^ for the wild-type. Shi et al. (2021) did a similar adaptation experiment but in the presence of oxygen. This study revealed that it is possible to adapt *S. ovata* to the presence of up to 5% oxygen and that *S. ovata* adapted to 0.5% oxygen produces 207.8 ± 17.5 mmol acetate m^–2^ day^–1^ which is 1.5-fold higher than the wild-type strain. This is of special importance because a slight oxygen tolerance will be advantageous in an industrial application and handling. There is oxygen produced on the anode which can leak to the cathode region and can cause problems for the anaerobic *S. ovata*. Indeed, even in the studied H-cells, the measured oxygen concentration in the cathode compartment ranged from 0.26 to 0.47 % in the time course of 120 h (Shi et al., 2021).

*Sporomusa ovata* does not seem to be a good biofilm builder on electrode surfaces. Nevertheless, different studies improved the attachment of the cells to the cathode and reported enhanced acetate production rates (Chen et al., 2016; Krige et al., 2021).

### Electrode Materials

**FIGURE 4:**
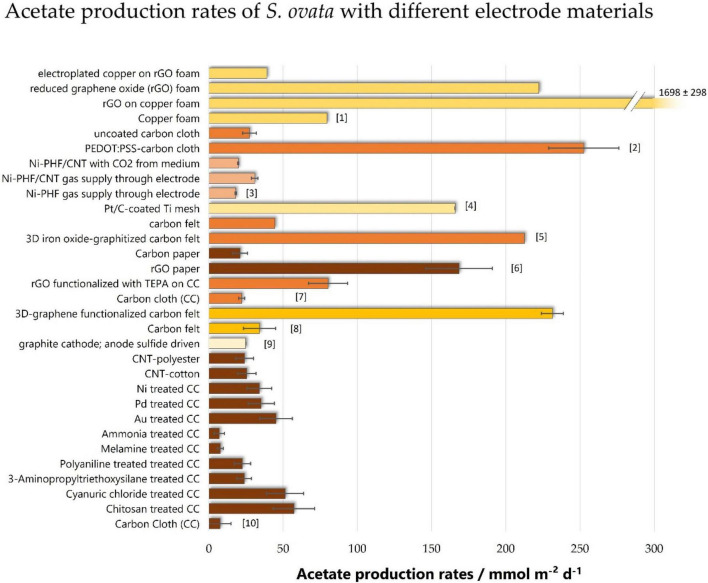
Acetate production rates of *S. ovata* with different electrode materials. Bars of the same color are derived from the same studies: [1]: (Aryal et al., 2019), [2]: (Aryal et al., 2016), [3]: (Bian et al., 2018), [4]: (Li et al., 2018), [5]: (Cui et al., 2017), [6]: (Aryal et al., 2017a), [7]: (Aryal et al., 2018), [8]: (Aryal et al., 2016), [9]: (Gong et al., 2013), [10]: (Zhang et al., 2013). On that basis, the same color results are directly comparable, as other parameters (cathode potential, medium, temperature, reference for normalization) are equal. rGO, reduced graphene oxide; PEDOT:PSS, poly(3,4-ethylenedioxythiophene:polystyrene sulfonate); Ni-PHF, porous nickel hollow fiber; TEPA, tetraethylene pentaamine; CNT, carbon nanotubes; CC, carbon cloth.

So far, one of the biggest advances in terms of acetate production rates was achieved through the optimization of electrode materials (Figure 4). They have a decisive impact on the cell-electrode interface and are crucial for a functioning biocatalyst. Generally, the most important parameters regarding electrode materials relate to distinct hierarchical levels: the electrode surface material itself, the electrode microstructure, and the electrode topology. While the surface is important regarding its chemistry, the electrode microstructure influences the electrochemically accessible surface area and the mass transport. The electrode topology on the other hand is the physical macrostructure and affects the electronic and ionic conductivity, the surface area per volume, and the mass transport (Kerzenmacher, 2017). Studies reporting different electrode materials often do not distinguish between these levels, making the interpretation difficult. For example, the coating of electrodes can increase accessible surface area and surface chemistry at the same time. This makes it difficult to distinguish if a bigger area or the surface chemistry leads to a different result.

#### Surface Chemistry

One unambiguous finding regarding the material chemistry is that providing a positive surface charge can effectively enhance MES (Zhang et al., 2013). The surface charge of *S. ovata* has a Zeta potential of about -12.8 ± 1.6 mV (Chen et al., 2016), which explains why the positive charge helps the bacteria to grow on the electrode. Coating a thin layer of chitosan on top of graphite felt provides a positive charge and Zhang et al. (2013) showed that this significantly enhances acetate production rates by 7.6-fold compared to uncoated graphite felt. This improvement comes along with 9× higher cell densities of *S. ovata* on the electrode surface and it was the highest improvement compared to other electrode modifications tested in this study. As the specific surfaces are not reported and the acetate production rate is normalized to the geometric area, it is not always clear to which extent the chemical modification, or the better microstructure is the reason for improvement.

#### 3D Electrodes

3D electrodes are described as a promising concept to increase current densities in all bioelectrochemical systems and target the improvement of the macrostructure. They specifically increase the area for bacteria to interact with the electrode which makes them extremely promising. Unfortunately, most studies with 3D electrodes are normalized to the geometric 2D surface, making comparison difficult.

Aryal et al. (2019) developed a copper foam coated with reduced graphene oxide (rGO), that allowed the production of 1,697.6 ± 298.1 mmol m^–2^ day^–1^ of acetate at -990 mV vs. SHE. This is by far the highest production rate with *S. ovata* and also any other pure culture reported in the literature. The copper core, which is highly conductive and inexpensive, provides an optimal carrier for the biocompatible rGO. Compared to the uncoated copper foam, the surface was increased 161-fold, while current density and acetate production rate were increased 3-fold and 21-fold, respectively. This condenses in very different coulombic efficiencies: While the uncoated copper displays a very low coulombic efficiency of 10 ± 6.5%, coating it with rGO results in 70.2 ± 14.1%.

Cui et al. (2017) investigated the acetate production of *S. ovata* with a three-dimensional hierarchical metal oxide–carbon electrode. This modification of a carbon felt electrode results in a 4.8-fold increase of acetate production rate. This study is the only one cited here that reports the acetate production normalized to the volume of the electrode instead of the geometric area.

To compare these volumetric production rates with other studies (Figure 4), the acetate production reported in Cui et al. (2017) was normalized to the reported size of the electrode’s geometric surface. However, we want to emphasize and encourage the shift of paradigm in future work to report production rates and current densities normalized to the volume of the electrode whenever it is not possible to completely rule out that the inner part of the electrode is accessible. Unfortunately, the volumetric current density or the accessible surface area is often difficult to assess from the limited data reported in the literature making it impossible to recalculate the values normalized to the volume or specific surface for all studies.

#### Gas Supply Through Electrodes

Another promising and exciting approach to increasing MES’s efficiency is the use of electrically conductive gas permeable materials as electrodes (Bian et al., 2018) or gas diffusion cathodes (Bajracharya et al., 2016). In this setup, the electrode itself acts as a three-phase boundary bringing together the four necessary components for acetate production: the substrate CO_2_, the bacteria as the catalyst, the medium providing H^+^, and the cathode delivering electrons. The direct supply of CO_2_ through the porous electrode showed a 1.6-fold increase in acetate production (Bian et al., 2018).

### Operation Parameters

Temperature, medium composition, and different cell voltages were also tested with *S. ovata* and are discussed in the following sections. But the pressure inside the reactor which influences gas solubility has not been investigated with *S. ovata*. The hypothesis that increasing pressure might be an easy way of optimizing MES (Jourdin and Burdyny, 2020) needs to be tested (Jourdin and Burdyny, 2020). Exemplary, pressurized reactors are known to improve the activated sludge process treating wastewater by increasing the dissolved oxygen levels (Jin et al., 2010). But we did not find MES studies with *S. ovata* exploring this possibility. In contrast, it was shown, that higher pressure generally slows down *S. ovata* growth (Sakimoto et al., 2014). Still, pressurizing the reactors would enhance the CO_2_ availability (Jourdin and Burdyny, 2020) and can therefore be an easy way of improving acetate production. The investigation of the balance between the two effects may reveal conditions that enhance the acetate production rate.

Another topic that has not been systematically investigated is the method of CO_2_ supply into the system. The effect of parameters like the volumetric flow of the gas, the gas composition, and reactor mixing on the acetate production rates of *S. ovata* is not known yet. In mixed community biofilms, it was shown that the supply of CO_2_ is favorable compared to HCO_3_ (Mohanakrishna et al., 2016; Izadi et al., 2020). Still, the chemical equilibrium between CO_2_ and HCO_3_ is determined by the pH. Thus, the form of carbon supply itself might be secondary compared to the pH in the system.

#### Temperature

**FIGURE 5:**
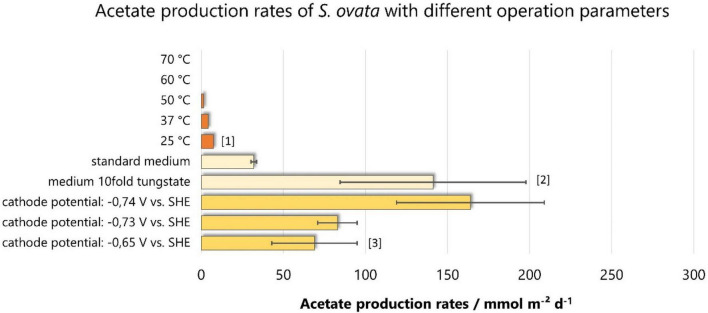
Acetate production rates among different operation parameters with *S. ovata.* Bars of the same color are derived from the same studies: [1]: (Faraghiparapari and Zengler, 2017), [2]: (Ammam et al., 2016), [3]: (Giddings et al., 2015). On that basis, the same color results are directly comparable, as other parameters (electrode material, reactor setup, Sporomusa strain, etc.) are equal.

The temperature is an essential parameter when trying to optimize MES. On the one hand, the gas solubility is higher at lower temperatures. On the other hand, the cells have an optimal temperature for growth. A study that compares different temperatures (Figure 5) showed that *S. ovata* achieves the optimal acetate production rate at 25°C, but no temperatures lower than this were investigated (Faraghiparapari and Zengler, 2017). This temperature is surprising, as 37°C, also tested in this study, is the temperature closer to the optimal growth temperature for *S. ovata* (between 34 and 39°C; Moeller et al., 1984). The higher acetate production rate at lower temperatures is likely to be related to the better gas solubility at lower temperatures. More fundamental research would be necessary to find out if the gas solubility of the possibly occurring H_2_ or of the substrate CO_2_ is decisive.

#### Medium Composition

The medium composition is also an essential factor influencing acetate production with *S. ovata*. Indeed, tungstate concentration in the medium influences the acetate production rates (Ammam et al., 2016). A 10-fold increase of tungstate concentration in the medium resulted in a 1.2-fold lower doubling time. The effect on acetate production is even higher: it increases the rate 4.4-fold (from 32.0 ± 1.7 to 141.2 ± 56.6 mmol m^–2^ day^–1^; Ammam et al., 2016). Analyzing the gene expression at different tungstate levels showed that enhanced biosynthesis of acetate and other compounds come along with higher gene expression of tungsten-containing aldehyde ferredoxin oxidoreductases and a tungsten-containing formate dehydrogenase. An increase in tungstate concentration also steers the process to ethanol production in *S. ovata* (Ammam et al., 2016).

#### Cathode Potential

Another decisive parameter affecting acetate production is the cathode potential. The more negative it is the higher are acetate production rates (Giddings et al., 2015; Tremblay et al., 2019). Unfortunately, it is not yet clear to what extent *S. ovata* performs direct electron uptake on the cathode or relies on the intermediate H_2_. In both cases, the level of knowledge hypothesizes that a low cathode potential is favorable, providing a higher driving force. But from the microbial perspective, the optimal electrode potential can depend on the used metabolic pathway and must be investigated. From a techno-economic assessment, the optimal cathode potential will be a trade-off between high acetate production rates and the needed energy to power low cathode potentials.

Giddings et al. (2015) report acetate production rates of *S. ovata* at different cell voltages resulting in cathode potentials of -0.74, -0.73, and -0.65 V vs. SHE. These results allow a first impression of the effect of cathode potential on the acetate production rates but do not draw a comprehensive picture. This study aims to implement a simple reactor setup without potentiostatic control and works with a DC power source between working and counter electrodes (cathode and anode, respectively). Thus, the resulting cathode potentials are not fixed and regulated but result from the imposed cell voltages. The difference of 10 mV in the resulting cathode potential between the reactors running at 3.5 and 5 V cell voltage can hardly explain the significantly higher acetate production rate of 164 ± 45 mmol m^–2^ day^–1^ at -0.73 V vs. SHE compared to 83 ± 12 mmol m^–2^ day^–1^ at -0.74 V vs. SHE. We assume that this correlation is not caused by the slight potential difference and want to point out that the influence of the cathode potential on MES performance with *S. ovata* is a critical knowledge gap to be studied.

### Microbial Electrosynthesis Process Engineering

*Sporomusa ovata* is not only used for acetate production directly on electrodes but was also already investigated in a two-stage reactor, where hydrogen is produced electrochemically on the electrode, which is then subsequently used by *S. ovata* to reduce CO_2_ to acetate. Kracke et al. (2019) report a maximum acetate production rate of 4.84 × 10^–3^ mmol h^–1^ with *S. ovata* and a 1 cm^2^ flat MoS_2_ electrode at ca. -1.0 V vs. SHE. The acetate production corresponds to 1,162 mmol m^–2^ day^–1^, which exceeds all reviewed acetate production rates of *S. ovata* with 2D electrodes (see Figure 4). Additionally, it has a very high coulombic efficiency reported at 103.7%. This value >100% indicates that a two-stage approach aiming and optimizing for intermediate hydrogen production is favorable. But a one-stage process circumventing hydrogen production with direct electron uptake of the biocatalyst will be more competitive in terms of safety and effectiveness (Rabaey and Rozendal, 2010). Another successfully tested concept is the introduction of hydrogen carriers into a system where hydrogen is produced abiotically (Rodrigues et al., 2019). With the help of the carrier, which in this case was a perfluorocarbon nanoemulsion, the hydrogen availability is improved in the entire reactor and acetate production rates increased by 190% could be shown (Rodrigues et al., 2019).

However, to conclusively assess this core question and decide which way to go, the biocatalyst’s underlying electron transfer mechanisms and energetic metabolism need to be investigated.

Independently of whether *S. ovata* uses direct or indirect electron transfer, the produced acetate will need to be extracted from the MES reactor. For this, Gildemyn et al. (2015) propose a three-compartment reactor system in which an anion exchange membrane and a cation exchange membrane separate the cathode and the anode compartments, respectively, (Gupta et al., 2019). In the resulting extraction compartment in the middle, the acetate spontaneously accumulates, driven by the electric field of the MES reactor.

And while acetate can be the target product, it can also be directly upgraded to long-chain alkyl esters in MES, aiming at the production of biofuels. Lehtinen et al. (2017) use *S. ovata* to produce acetate, and *Acinetobacter baylyi* subsequently converts it into long alkyl esters in a second aerobic step (Lehtinen et al., 2017). *S. ovata* is also capable of producing ethanol (Blanchet et al., 2015; Ammam et al., 2016), and 2-oxobutyrate (Nevin et al., 2010). These products are not in the scope of this review but demonstrate the versatility of *S. ovata* for different processes.

## Electron Uptake Mechanisms of *S. ovata*

In the case of mixed communities in MES, there is evidence that biofilms have paramount importance for electron uptake and acetate production (Jourdin et al., 2016; Izadi et al., 2020). This biofilm role also seems true for pure *S. ovata* cultures: Aryal et al. (2016) showed that a dense immobilized biofilm in a bioinorganic matrix significantly improves current densities and acetate production rates. But a systematic study of the biofilms‘ contribution to current uptake compared to the planktonic fraction is not yet available. This open research question is interdependent with the direct or indirect nature of electron uptake by *S. ovata*. While Nevin et al. (2010) stated that *S. ovata* consumes electrons directly from an electrode, newer publications suggest a hydrogen-dependent electron uptake process (Tremblay et al., 2019). To assess possible electron uptake mechanisms, multiheme cytochromes and hydrogenases were searched in the genome of *S. ovata* DSM 2662 (GCF_000445445.1) using Interproscan 5.39–77.0. Only matches that were simultaneously present in the RefSeq (NCBI) and UniProtKB databases were considered.

### Indirect Electron Uptake *via* Hydrogen

If the electron uptake occurs indirectly through hydrogen, hydrogen must be formed by reducing water. Hydrogen can then be used as the electron donor in *S. ovata* to form acetate with CO_2_. Different ways of hydrogen evolution are possible: (i) abiotic production at electrode potentials ≤-500 mV vs. NHE (Karthikeyan et al., 2019); (ii) hydrogen evolution at higher electrode potentials ≤-300 mV vs. NHE through the catalytically active and secreted compounds like hydrogenases but of unknown nature; (iii) abiotic hydrogen production at higher electrode potentials as expected due to very low partial pressures of hydrogen (Philips, 2020).

The difficulty in exploring the role of hydrogen as an intermediate is that hydrogen is consumed by the bacteria at a fast rate, making the detection difficult. Tremblay et al. (2019) placed a microsensor close to the cathode, which could not detect hydrogen evolution at -501 mV vs. NHE with the abiotic fresh medium. Interestingly, in cell-free but already used medium by *S. ovata*, hydrogen production was observed at more positive potentials (-301 mV vs. NHE; Tremblay et al., 2019). This observation suggests the presence of secreted metabolites, cell components, or chemicals produced by *S. ovata* which enhance the catalytic hydrogen production. Indeed, Co and Ni deposition on the cathodes was determined, but no intact hydrogenases were found. Theoretically, it is possible that *S. ovata* secretes hydrogenases or uses hydrogenases on the cell membrane to form hydrogen which will subsequently be used as the electron donor. A search of the genome and annotated proteins reveals at least four different hydrogenase complexes coded. Among them are [NiFe] and [FeFe] hydrogenases, whose contribution to MES would need to be investigated. Table 1 lists all found hydrogenases. Still, there is no evidence to prove if hydrogenases in *S. ovata* are secreted or acting intracellularly.

**TABLE 1:** Annotated hydrogenases of *S. ovata* 2662.

Annotation		UniProtKB/RefSeq identification	Sequence length
[NiFe] hydrogenase	Small subunit	A0A0U1KYU5/WP_021167301.1	376
	Large subunit	A0A0U1KYU3/WP_021167302.1	630
[NiFe] hydrogenase	Small subunit	A0A0U1KUF0/WP_021169774.1	411
	Large subunit	A0A0U1KUH9/WP_021169773.1	458
Periplasmic [Fe] hydrogenase	Small subunit	A0A0U1KSK8/WP_021169148.1	103
	Large subunit	A0A0U1KSM2/WP_021169149.1	419
[NiFe] hydrogenase		A0A0U1L3V7/WP_021170382.1	454
Periplasmic [Fe] hydrogenase		A0A0U1KV30/WP_040763082.1	581

*Searched by annotations via UniProt.*

Visser et al. (2016) introduce an *S. ovata* strain called An4, for which the authors report a [NiFe] hydrogenase complex for which the small subunit contains a tat signal motif. The presence of such a signal motif opens up the possibility that this hydrogenase (WP_021167303.1) is transported across the cytoplasmic membrane. It is also present in *S. ovata* 2662. Still, there is no evidence for hydrogenases, which are also transported across the outer cell membrane to the surrounding medium. This capability was previously reported for *Methanococcus maripaludis* (Deutzmann et al., 2015; Tsurumaru et al., 2018), and a mixed biofilm (Marshall et al., 2017) but never for *S. ovata*.

It has also been hypothesized that the hydrogen consumption by acetogenic bacteria induces a low partial pressure, which again favors hydrogen evolution by reducing the activation energy without additional catalysts (Philips, 2020).

Intracellular hydrogen formation, low hydrogen partial pressures, and immediate consumption make hydrogen detection challenging. To better understand the role of hydrogen, it is necessary to reveal the electron uptake mechanism on a biomolecular level.

### Hypothetical Mechanisms for Direct Electron Uptake

Multiheme *c*-type cytochromes play a decisive role in known electron transfer mechanisms, especially in extracellular electron transfer pathways of exoelectrogens that transfer electrons to an anode, including *Geobacter* and *Shewanella*. Similar pathways can function in reverse on the cathode in some electroactive organisms, such as *R. palustris* TIE-1 (Gupta et al., 2019). Although, for *S. ovata*, the existence of multiheme *c*-type cytochromes is reported (Kamlage et al., 1993; Visser et al., 2016), it is not yet known if they are involved in electron uptake processes (Table 2). Should such a mechanism be operative, it could feed electrons directly in the bioenergetics electron transport chain or promote intracellular hydrogen production *via* hydrogenases or nitrogenases (Marshall et al., 2017; Izadi et al., 2020) at unknown electrode potentials.

**TABLE 2:** *C*-type cytochromes of *S. ovata*, determined by a survey at UniProt, NCBI.

Annotation		UniProtKB/RefSeq identification	Localization prediction	Number of heme binding motifs	Sequence length
Cytochrome *c* nitrite reductase NrfHA complex (1)	NrfH	A0A0U1L5R5/WP_021171263.1	Periplasm (soluble)	4	157
	NrfA	A0A0U1L5U9/WP_021171262.1	Inner membrane	5	157
Cytochrome *c* nitrite reductase NrfHA complex (2)	NrfH	A0A0U1L5R7/WP_021171250.1	Periplasm (soluble)	4	157
	NrfA	A0A0U1L5Q8/WP_021171249.1	Inner membrane	5	427
Cytochrome *c* nitrite reductase NrfHA complex (3)	NrfH	A0A0U1KWH4/WP_021168864.1	Periplasm (soluble)	4	157
	NrfA	A0A0U1KXE4/WP_021168865.1	Inner membrane	5	429
Multiheme cytochrome/molybdopterin-binding oxidoreductase		A0A0U1L3K6/WP_021170960.1	Periplasm (soluble)	2	373

*The localization prediction was determined with https://www.psort.org/psortb/ and SignalP-5.0, http://www.cbs.dtu.dk/services/SignalP/.*

Nitrogenases fix dinitrogen to ammonia and co-produce H_2_, but are also capable of reducing other substances such as CO_2_ to formate. In the absence of these, nitrogenase will lead the electron flux toward hydrogen (Milton and Minteer, 2019), meaning that electrons are accepted by the cell and transported to the nitrogenases. If the electrons are received directly from the electrode or through a carrier needs to be discussed. In any case, hydrogen is produced inside the cells. If the hydrogen gets released into the medium, it would then contribute to indirect electron transport and electrons stored in the hydrogen could be reused. Depending on the presence of nitrogen species in the medium, different amount of hydrogen gets produced. According to the reaction stoichiometry of N_2_ reacting to NH_3_ catalyzed by nitrogenases, the produced hydrogen contains 25 % of the originally up taken electrons (Hoffman et al., 2009).

Marshall et al. (2017) showed that the nitrogenase encoding NifH is one of the most abundant genes in a mixed community biofilm with high acetate production rates, suggesting an essential role in acetate production on cathodes. An analysis of the *S. ovata* 2662 genome in the PATRIC resource center^1^ and UniProt databank reveals the presence of NifH, NifB, NifE, NifN, and NifDK encoding for FeMo nitrogenases. The presence of these genes is an indication that this route of transient hydrogen production might also play a role in acetate production with *S. ovata*. Interestingly, but in opposite direction, Tremblay et al. (2015) show that a highly efficient methanol adapted strain of *S. ovata* producing acetate, had downregulated the gene SOV_1c07070 which is annotated as a dinitrogenase FeMo cofactor biosynthesis protein. But this might happen since the medium used in this study contains ammonium chloride resulting in transcriptional downregulation of the redundant nitrogenase as a strategy to save energy.

A close look into the proteome of *S. ovata* reveals that 6 out of 7 *c*-type cytochromes belong to NrfHA complexes attributed to the nitrite reductase family (determined by UniProt; Igarashi and Kato, 2021). These are membrane-attached proteins that agree with the reported presence of *c*-type cytochromes in the membrane fractions of *S. ovata* (Moeller et al., 1984). Indeed, the BLAST survey on NCBI reveals homology with the well-characterized NrfHA complex of *Desulfovibrio vulgaris*, which is bound to the periplasmic membrane (Rodrigues et al., 2006; Luisa Rodrigues et al., 2008). All three encoded NrfAH complexes are paralogs. Another *c*-type cytochrome is the WP_021170960.1 which does not show homology to any known protein and thus remains hypothetical. The sequence reveals that it is a soluble dihemic protein, which can be involved in electron transfer in the periplasmic space of *S. ovata*.

## Concluding Remarks

From the previous sections, we can conclude that *S. ovata* is a very promising biocatalyst for the generation of acetate in MES. And considerable work has already been done for optimizing cathode materials, operation parameters, and microbiological adaptation. The achieved acetate production rates with *S. ovata* are far beyond other species, and ALE experiments showed the flexibility of *S. ovata* to increase acetate production and oxygen tolerance by adaptation. But still, the net acetate production rates obtained need to be improved to drive MES into application scenarios. Fruehauf et al. (2020) rate the technological readiness level (TRL) among different products and different MES configurations and conclude that it is TRL 2-3 for biofilm-based MES and TRL 4-5 in two-stage reactors with hydrogen as intermediate. TRL 6 is considered to be the transition point from research to industrial implementation. Among others, one significant knowledge gap and research needed to push these technologies to the next development levels is the understanding of the underlying electron uptake mechanisms. To further optimize systems running with *S. ovata* and aiming for acetate production, it is of utmost importance to reveal if current uptake happens directly on the electrode or if *S. ovata* can steer MES indirectly through enzymes improving the intermediate hydrogen production. The latter is expected to be driven through hydrogenases or nitrogenases but has not been elucidated. The study of cathode potentials and their effect on the acetate production of *S. ovata* is also lacking and would already point out the underlying nature of direct or indirect electron transfer.

Another area to be studied is the role of nitrogenases in MES. Practically it is crucial to assess if the presence of nitrogen in gaseous or ammonia form influences the outcome of MES. To our best knowledge, this parameter has not yet been investigated.

On the protein level, it is essential to understand if *c*-type cytochromes are involved in the current uptake and, in that case, to reveal their function. To address these open issues, it is necessary to develop strategies to manipulate *S. ovata* genetically. Until this is possible, the production and characterization of the individual proteins provide an alternative to characterize their function (Louro et al., 2018). There are good prospects that *S. ovata* can be made genetically tractable by adapting tools developed for *C. ljungdahlii* described in Molitor et al. (2016) as *S. ovata* shows considerable genetic similarities to Clostridia. In general, genetic engineering will be of utmost importance and of high value to answer these fundamental questions about the electron uptake mechanisms on cathodes.

Making a back-of-the-envelope calculation summing up all achieved improvements in the discussed biological, operational and engineering parameters could improve acetate production rates more than 80 times. Summing up the enhancements which rely on different parameters in different studies is not a rigorous way to assess the great potential of *S. ovata*. Still, it provides an estimate of the magnitude of the challenge ahead. A better understanding of the nature of the underlying electron transfer will allow assessing the bottlenecks of MES and will be the next step on the way to application.

## Author Contributions

JM: conceptualization, formal analysis, investigation, writing – original draft, writing – review and editing, visualization, project administration, and funding aquisition. CP: investigation, writing – original draft, writing – review and editing, visualization, and supervision. RS: investigarion, data curation, and visualization. RL: conceptualization, validation, resources, writing – review and editing, and supervision. All authors contributed to the article and approved the submitted version.

## Conflict of Interest

The authors declare that the research was conducted in the absence of any commercial or financial relationships that could be construed as a potential conflict of interest.

## Publisher’s Note

All claims expressed in this article are solely those of the authors and do not necessarily represent those of their affiliated organizations, or those of the publisher, the editors and the reviewers. Any product that may be evaluated in this article, or claim that may be made by its manufacturer, is not guaranteed or endorsed by the publisher.

## Footnotes

1
patricbrc.org
